# Ceruloplasmin and Ferritin Changes in Ocular Fluids from Patients with Vitreoretinal Diseases: Relation with Neuroinflammation and Drusen Formation

**DOI:** 10.3390/ijms26136307

**Published:** 2025-06-30

**Authors:** Graziana Esposito, Pamela Cosimi, Bijorn Omar Balzamino, Marisa Bruno, Rosanna Squitti, Lucia Dinice, Fabio Scarinci, Mauro Ciro Antonio Rongioletti, Andrea Cacciamani, Alessandra Micera

**Affiliations:** 1 Research and Development Laboratory for Biochemical, Molecular and Cellular Applications in Ophthalmological Science, IRCCS—Fondazione Bietti, 00184 Rome, Italy; graziana.esposito@fondazionebietti.it (G.E.); bijorn.balzamino@fondazionebietti.it (B.O.B.); lucia.dinice@fondazionebietti.it (L.D.); 2 Surgical Retina Research Unit, IRCCS—Fondazione Bietti, 00184 Rome, Italy; pcosimi@gmail.com (P.C.); marisa.bruno@fondazionebietti.it (M.B.); fabioscarinci@gmail.com (F.S.); andrea_cacciamani@hotmail.com (A.C.); 3 Department of Laboratory Science, Research and Development Division, Ospedale Isola Tiberina—Gemelli Isola, 00186 Rome, Italy; maurociroantonio.rongioletti@fbf-isola.it; 4 Department of Theoretical and Applied Sciences, eCampus University, Viale Massenzio Masia, 26, 22100 Novedrate, Italy

**Keywords:** ceruloplasmin, ferritin, vitreoretinal diseases, epiretinal membranes, macular holes, aqueous, vitreous, Alzheimer’s disease, full-thickness macular holes

## Abstract

This pilot study explored whether the ceruloplasmin (CP) and ferritin (FT) levels in ocular fluids could serve as biomarkers for early neurodegenerative diseases (Alzheimer’s, Parkinson’s, and other dementias). CP and FT are known to modulate neurodegenerative tissue responses. We analysed aqueous and vitreous samples from 26 patients (8M/18F, aged 60–85) who were undergoing elective vitreoretinal (VR) surgery. Of these, 14 had idiopathic epiretinal membranes (ERMs), 6 had idiopathic macular holes (MH), and 6 were patients with Alzheimer’s disease (AD) who presented with VR disorders (VRDs). CP, FT, and selected neuroinflammatory mediators such as interferon γ (IFN-γ), interleukin (IL-6), vascular endothelial growth factor (VEGF), nerve growth factor (NGF), and brain-derived neurotrophic factor (BDNF) were quantified. Odds ratio analysis was applied to assess the CP/FT ratio’s association with subretinal drusen. We found distinct CP and FT profiles in VRD samples. In aqueous fluid, the CP increased and the FT decreased in early-stage ERM, which reduced the CP/FT ratio. Similar patterns were observed in vitreous fluid. The CP levels correlated with the VEGF (aqueous), IL-4 (vitreous), NGF, and BDNF levels; FT correlated with IL-6 and NGF. A higher CP/FT ratio was associated with increased risk for neurodegenerative conditions. Our findings support the quantification of CP and FT in ocular fluids as a promising approach for identifying early neurodegenerative changes and suggest that the CP/FT ratio may be linked to drusen imaging and clinical neurodegenerative history.

## 1. Introduction

Ocular surgery and, particularly, macular surgery have undergone a remarkable technological evolution due to the increasing demand of therapeutic surgery in industrialized countries and the advent of the use of artificial intelligence in surgery [1]. Vitreoretinal (VRD) surgery approaches can restore, preserve, and even enhance the vision in patients suffering from macular puckers, macular holes, and a detached retina [2,3,4]. An epiretinal membrane (ERM) is an avascular, fibrocellular membrane layered at the vitreoretinal interface, whereas a macular hole (MH) is a small hole in the macula itself [5]. ERMs, MHs, and age-related macular degeneration (AMD) are characterized by Drusen formation and represent common manifestations of “old to elder” macula evolution [6,7,8].

In recent years, corroborating data sustain the neuro-ophthalmological implications of several neurodegenerative diseases, prospecting the possibility of identifying common ocular biomarkers for the differential diagnosis of not-yet-diagnosed Alzheimer’s disease (AD), Parkinson’s disease (PD), senile dementia (SD), or metabolic neurodegenerative diseases (diabetes) [9,10]. Reinforcing this, the widespread Alzheimer precursor protein (APP) and mature β-Amyloid (Aβ) were detected not only in brain tissues/fluids but also in ocular tissues from deceased humans and experimental AD (transgenic mice) [11]. Aβ deposits layered over the retinal pigmented epithelium and inside the inner retina, merely in association with retinal neuron loss within the ganglion cell layer, inner plexiform layer, inner nuclear layer, and retinal fiber layer, were observed in postmortem human and AD murine retinas [12,13]. Furthermore, Aβ was found in cataractous lenses and tested as toxic for cultured human lens cells [14]. Of interest, Gharbiya et al. and An et al. described the presence of Aβ in tears from patients with AD and correlated it with choroidal thickness and AD severity, prospecting human tears as a good alternative biological matrix for detecting “early” AD markers [15,16].

Over the past decade, growing evidence has confirmed the role of essential trace metals in the onset and progression of AD, particularly implicating excess “free” copper—defined as the fraction of serum copper that is not bound to proteins, which is primarily ceruloplasmin (CP), and known also as non-ceruloplasmin copper—and abnormal iron deposition in cognitive decline [17,18]. Aβ monomers may exacerbate tissue aging and neuronal loss by generating oxidative stress mediators, a process that is partially mediated by their metal-binding properties [19,20]. As free copper accumulates in the brain, a systemic imbalance emerges between reduced protein-bound copper, which serves as a cofactor in various physiological processes, and elevated levels of unbound, toxic copper, potentially leading to significant neurodegenerative damage [21]. Circulating unbound copper discriminates between AD and healthy elderly groups, and between mild cognitive impairments (MCIs) and healthy controls, but does not discriminate between AD and MCIs [22]. Functionally, CP and cytosolic/serum ferritin (FT) are enzyme and carrier proteins for copper and iron, respectively [23]. The link between these two glycoproteins lies in the fact that CP is a key iron oxidase in catalyzing the reaction from divalent to trivalent iron and promotes iron binding to transferrin protein [23]. Despite the increasing amount of literature on the topic, much is still unknown about the exact functions of these systemic and cytosolic proteins, as well as their crosstalk upon pathological states (senescence, ageing, and age-related disorders linked to cognitive decline and dementia).

Building on our recent review discussing the interplay between copper, iron vitreoretinal disorders (VRDs), and subretinal drusen imaging—and considering that both copper and iron have been successfully quantified in biological fluids of patients with AD—we sought to investigate whether the copper-binding protein (CP) and the iron-storage protein ferritin (FT) could also be reliably detected in ocular humors collected during VRD surgery. More specifically, our working hypothesis is that measurable levels of CP and FT in ocular fluids may correlate with specific vitreoretinal pathologies, particularly ERM and MH, and thereby reflect underlying neurodegenerative or inflammatory mechanisms [24]. To do so, we quantified the CP and FT in aqueous and vitreous biological samples from VRD that were collected at the time of phaco-vitrectomy and then conducted the following: i. we assessed differences between ERM or MH subgroups; ii. we studied the association of CP and FT expression with that of a few selected inflammatory/neurodegenerative mediators that are representative of inflamed status; and iii. we verified the association of CP and FT with drusen imaged by optical coherence tomography (OCT) and confirmed the selective expression in established AD VRD subjects.

## 2. Results

Twenty-six (8M/18F) patients were included in this observational one-point case-control study, and recruitment was carried out according to inclusion and exclusion criteria (see Materials and Methods). The demographical and preoperative bioinstrumental data are summarized in Table 1. Briefly, the mean age of the entire population was 69.6 ± 6.71, ranging from 60 to 85 years, with merely 70.0 ± 6.6 having ERMs and 68.7 ± 7.0 having MHs (*p* = 0.385). At the screening visit, the patients were interviewed, with their previous and current non-neurological (hypertension, cardiovascular), metabolic (diabetes, thyroiditis), and neurological (AD, PD, senile dementia (SD)) comorbidities being documented. Patient-reported comorbidities are shown in Table 1. Particularly, some patients referred to familial hypercholesterolemia and/or statin therapy (11/26). This parameter was also registered since high cholesterol represents one of the main risk factors for developing neurological diseases. From this initial screening, an additional subgroup was created, including VRD subjects with a diagnosis of AD (*n* = 6, 70.0 ± 6.6). After surgery, the collected samples were sub-grouped, according to anamnesis and bio-instrumental assessment, as either ERM or MH. For statistical analysis, two main groups, ERMs and MHs, were created, and the ERM group was sub-grouped into stage 2, stage 3, and stage 4, depending on the OCT findings and the adoption of Govetto’s staging classification system [25]. The AD subgroup was tested to confirm the initial hypothesis.

**Figure 1:**
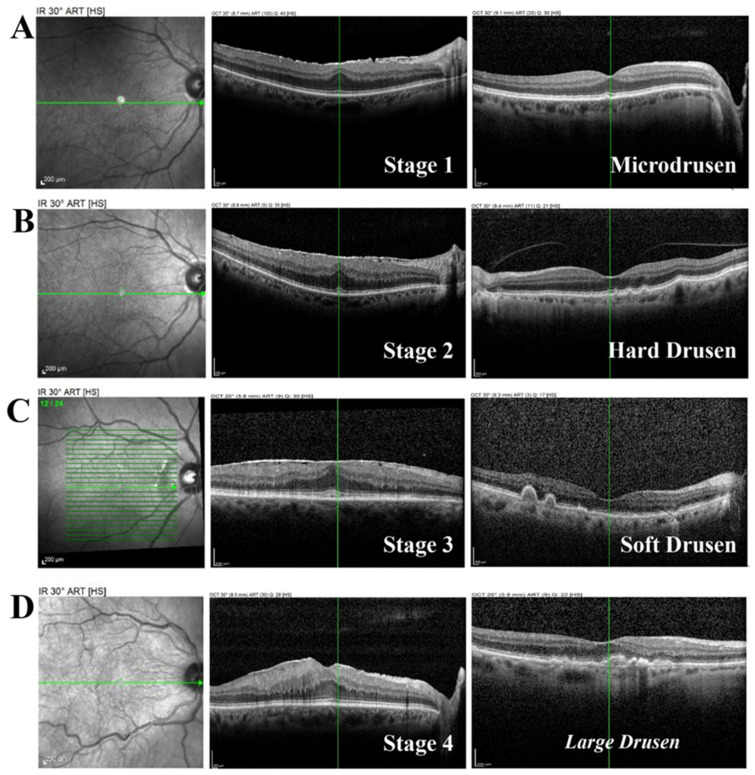
AD Structural findings of preoperative retinas. Representative spectral domain OCT-associated images (SD-OCTs) from eyes of subjects who underwent coupled cataract-vitreoretinal surgery. The condition of retinal layers in ERM-affected eyes, and drusen formation at the macular area, are staged as follows: (**A**), stage 1; (**B**), stage 2; (**C**), stage 3; (**D**), stage 4. Note that control was omitted while stage 1 was displayed, although this stage was not used for the biomolecular analyses. From left to right: fundus, retinal layers, and schematic representation of drusen. Note: micro drusen (**A**); hard drusen (**B**); soft drusen (**C**); large drusen (**D**). Green layers: drusen, peripheral drusen, and macular-positioned large drusen formations.

As displayed in Figure 1, OCT imaging was conducted to determine the presence of micro- and macro-drusen in our study population.

Although the biochemical analyses were carried out only for the ERMs (stage 2, stage 3, and stage 4) and MHs, some micro drusen were also observed in the retinas of patients with stage 1 ERM. These patients were not included in our study population as they underwent no therapeutic surgery. The pre-surgery ophthalmological examination carried out with SD-OCT showed the presence of drusen formations in 15/20 cases, mainly localized at the choroidal-macular area. The images were subjected to digital analysis, and the drusen formations were counted, sized, and classified as either absent, micro drusen (Figure 1A), or macro drusen (clustered as hard (Figure 1B), soft (Figure 1C), and large (Figure 1D)). Although Figure 1 displays the presence of a micro drusen at stage 1, these samples were not available in the analytical phase, as stage 1 was not considered for therapeutic phaco-vitrectomy. Furthermore, the images displayed in Figure 1 are only representative of the states and do not imply, for instance, the presence of hard drusen exclusively in stage 2.

### 2.1. Ceruloplasmin and Ferritin Levels Can Be Measured in Ocular Fluids and Are Changed upon Disease

The phaco-vitrectomy represents the only way to access the aqueous and vitreous for analysis. Herein, both fluids were collected and stored according to standardized procedures. Age/sex matched control samples were also considered: the control aqueous was collected at the time of cataract surgery of “healthy” subjects, while the control vitreous was obtained from an internal repository source (2019-Lab Bietti) sustained by untouched biological rests from other studies, and both were named ctr.

**Figure 2:**
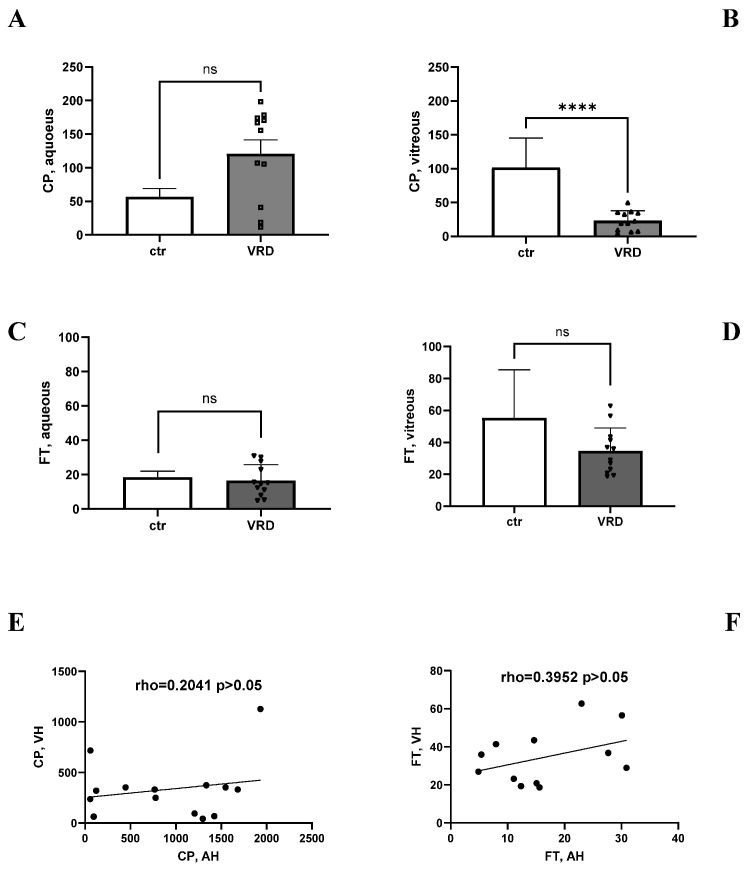
Ceruloplasmin and ferritin are detected in ocular fluids and are changed in these VRDs. Aqueous and vitreous were collected at the time of phaco-vitrectomy and cleared for ELISA assay. (**A**–**D**) The histograms show the different expressions of ceruloplasmin and ferritin in ocular humours from VRD cases. Note that ceruloplasmin levels were higher in aqueous (**A**) and lower in vitreous from VRD cases (**B**), while the levels of ferritin were unchanged in aqueous (**C**) and lower in vitreous from VRD cases (**D**). (**E**,**F**) A tendency to a positive correlation was observed between aqueous and vitreous ceruloplasmin and (**E**) between aqueous and vitreous ferritin (**F**). ANOVA Tukey-Kramer post hoc comparison (**A**–**D**) and Pearson correlation (**E**,**F**). Legend: VRD, vitreoretinal diseases; AH, aqueous humour; VH, vitreous. Note that ctr is control aqueous and vitreous samples coming from, respectively, healthy cataract surgery (aqueous) and from repository biosamples (normal vitreous). Specific *p* values are shown by asterisks (ns not significant or **** *p* < 0.001).

As shown in Figure 2, the CP and FT proteins were quantified in the aqueous and vitreous from all VRDs and compared to matched-control samples. The levels of CP were not significantly high in the aqueous (*p* > 0.05; Figure 2A) and were significantly low in the vitreous (*p* < 0.005; Figure 2B) of the VRD population, as compared to related controls. Also, the levels of FT were unchanged in the aqueous (*p* > 0.05; Figure 2C) and lowered in the vitreous (*p* > 0.05; Figure 2D) of the VRD cases as compared to controls. A slight positive correlation was observed between the aqueous and vitreous CP (rho = 0.2041, *p* = 0.4839; Figure 2E), and between the aqueous and vitreous FT (rho = 0.3952, *p* = 0.2036; Figure 2F). No significant changes (*p* > 0.05) were obtained considering the main non-neuronal comorbidities (hypertension, cardiac and circulatory diseases, hypercholesterolemia, diabetes, and thyroiditis).

### 2.2. CP and FT Levels Are Linked to VR Disease

ERMs and MHs differentially impact the macula. The fibro-cellular membrane in patients suffering from ERMs can pull and damage the retina even if it heals correctly upon a normal background, while it can heal differently in the presence of pre-existing local scarring or if vitreous shrinking is too hard [26]. Over time, the vitreous can pull away from the retina, causing tension and tears in the macula and the production of MHs, which is often considered as an “uninflamed” state [27]. An ERM can be associated with surface retinal traction, displaying lamellar macular changes, partial-thickness macular holes, and cystoid macular edema (CME), while ERMs rarely cause a full-thickness MH [28].

**Figure 3:**
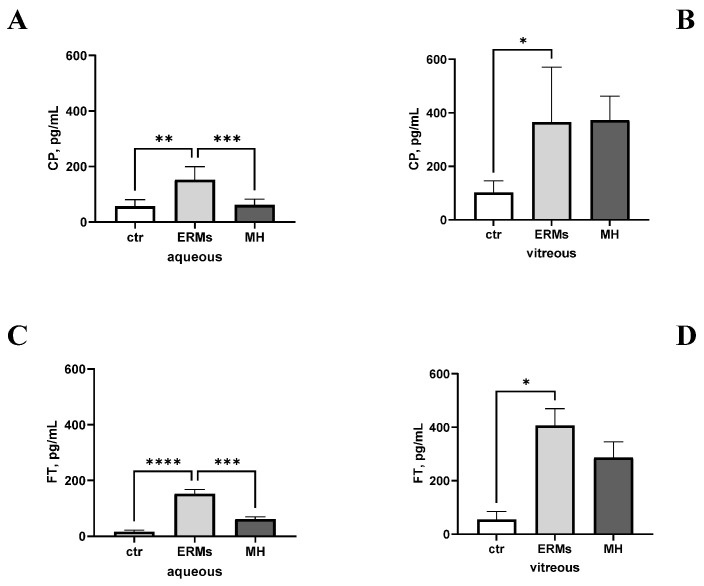
Differential CP and FT levels in ERMs and MHs. Histograms show the different levels of ceruloplasmin (**A**,**B**) and ferritin (**C**,**D**) in aqueous (**A**–**C**) and vitreous (**B**–**D**) fluid from ERMs and MHs. Note the increased ceruloplasmin levels in aqueous from ERMs with respect to MHs (**A**) and the lack of changes in the vitreous (**B**). The ferritin levels in the aqueous of ERMs were increased compared to that of MHs (**C**), and a slight increased expression of ferritin was seen in the vitreous of ERMs with respect to MHs (**D**). Note that both CP and FT levels in ocular humours were higher with respect to controls. ANOVA followed by Tukey-Kramer post hoc. Legend: CP, ceruloplasmin; FT, ferritin; ERM, epiretinal membranes; MH, macular holes. Specific *p* values are shown by asterisks (* *p* < 0.05; ** *p* < 0.01; *** *p* < 0.005 or **** *p* < 0.001).

Due to these functional and structural differences, aqueous and vitreous from ERMs and MHs were analysed and compared for their CP and FT protein expression. As shown in Figure 3, the aqueous CP levels were significantly higher in the ERMs than in the MHs, both with respect to the control groups (*p* < 0.00001; Figure 3A). By contrary, the vitreous CP levels were comparable between the ERM and MH subgroups, and were high with respect to the control groups (*p* > 0.05; Figure 3B). The FT levels were high in the aqueous from ERMs and MHs with respect to controls, and a higher expression was observed in ERMs than MHs (*p* < 0.05; Figure 3C). The FT levels were not significantly higher in the vitreous from the ERMs than in that from the MHs, and both were higher with respect to controls (*p* > 0.05; Figure 3D).

### 2.3. CP and FT Levels Are Linked to ERM Severity

At the cellular level, ERMs are scar tissue with a fibro-cellular texture that originates and layers over the retina, contracting and wrinkling the vitreoretinal interface. Retraction is driven by the local proliferation and activation of myofibroblastic cells and autocrine- and paracrine-sustained cells (tyrosine kinase receptor (trk), small mother against decapentaplegic (smad), extracellular signal-regulated kinases (ERKs), JUNK, and protein kinase B (AKT)-mediated pathways) [29]. Since, in other experimental models, CP overexpression inhibited the phosphorylation of some remodelling-associated signal proteins (Smad3, Erk1/2, p38, JNK, and AKT), while CP knockdown exerted an effect over phosphorylation, we hypothesized a direct involvement of CP in the functional activation of myofibroblastic cells populating the fibro-cellular membranes and regulating the contractile effects underneath the retina [30].

**Figure 4:**
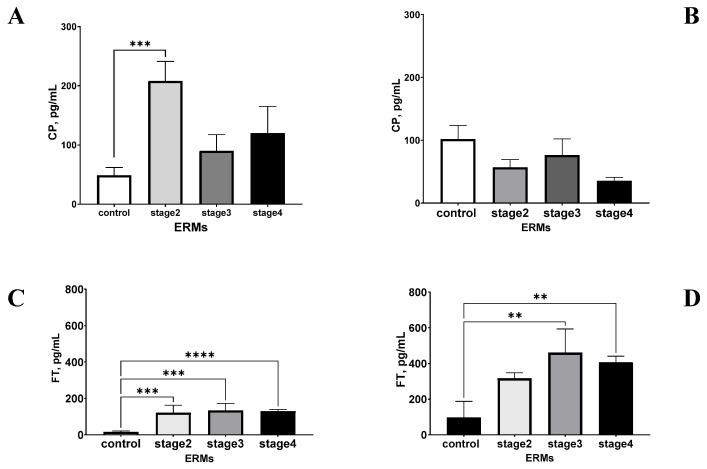
Ceruloplasmin and ferritin levels as a function of ERM severity. Histograms show the different expressions of ceruloplasmin (**A**,**B**) and ferritin (**C**,**D**) in aqueous (**A**,**C**) and vitreous (**B**,**D**) humours, respectively. Note the increased ceruloplasmin levels in aqueous from stage 2 ERMs (**A**), and a decreased vitreous ceruloplasmin content in stage 4 ERMs (**B**), both compared to their corresponding controls. A trend toward an increase was observed for aqueous (**C**) and vitreous (**D**) ferritin at all ERM stages. ANOVA followed by Tukey-Kramer post hoc. Legend: CP, ceruloplasmin; FT, ferritin; ERM, epiretinal membranes. Specific *p* values are shown by asterisks (** *p* < 0.01; *** *p* < 0.005 or **** *p* < 0.001).

Therefore, the staging system proposed by Govetto et al. was used for grading ERMs by their disease severity [25]. This clustering analysis showed that the CP levels were elevated across all stages of ERM; however, the increase was not proportional to the disease severity. Notably, the aqueous CP concentrations peaked significantly at stage 2, rather than progressively increasing, although they did not peak significantly with higher ERM grades (stages 3 and 4). (Figure 4A). On the contrary, the vitreous CP levels were lower in all ERM stages than in controls (Figure 4B). Notably, higher levels of FT were quantified in aqueous from ERMs, but the protein expression did not follow the ERM grading (Figure 4C). Also, high levels of ferritin were quantified in vitreous ERM fluid at all stages, with a slight peak at stage 3 (Figure 4D).

### 2.4. CP and FT Levels Are Not Linked to Systemic Inflammatory, Metabolic, and Neurodegenerative Disorders

There is little evidence that these neurodegenerative disorders are closely associated with long-lasting inflammation, reactive gliosis, or impairments of Aβ distribution and Tau cleavage inside neuronal tissues (brain, retina, and tears), although a dynamic protein signature from mild cognitive decline (MCD) to AD has been observed [31]. Inflammatory and neurodegenerative biomarkers were increased in both the ERMs and MHs.

**Figure 5:**
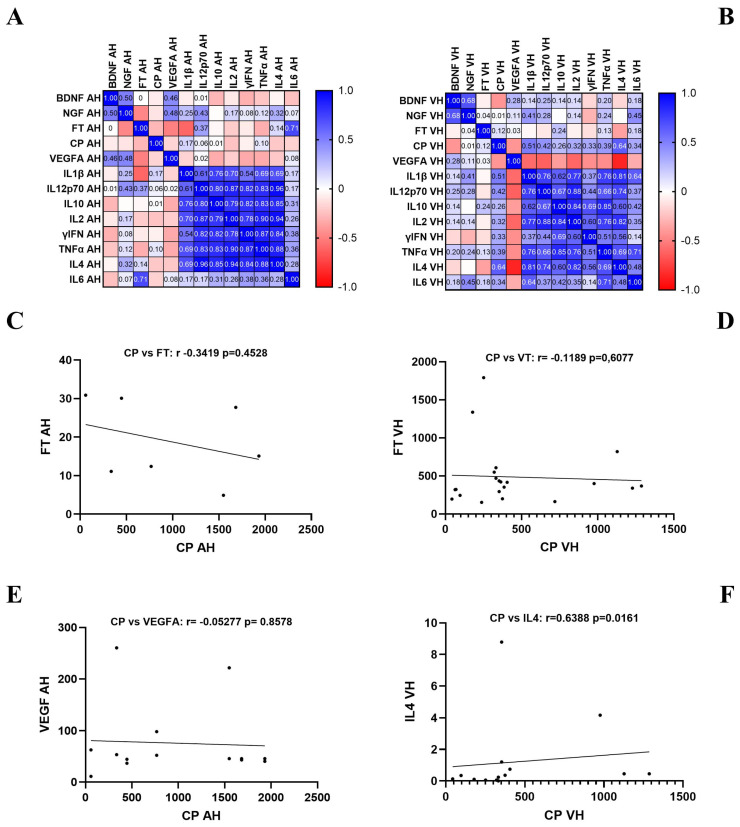
Differential biomarker expression in ocular fluids and specific correlations with ceruloplasmin and ferritin. Protein profiling was assessed considering the main targets representative of VRD diseases (60 selected biomarkers). (**A**,**B**) A correlation matrix is shown for both aqueous and vitreous. (**C**,**D**) A correlation rate was assessed for CP and FT in aqueous and vitreous. (**E**,**F**) A positive correlation was observed between CP and VEGF-A in AH, while an interesting correlation was obtained between CP and IL-4 in VH. Legend: CP, ceruloplasmin; FT, ferritin; AH, aqueous; VH, vitreous. Data are from protein microarray analysis (ANOVA followed by Bonferroni correction for n. targets) and were compared with Pearson r analysis (95% CI; α = 0.05).

As shown in Figure 5, the protein array analysis using a specific cluster of mediators known to be influenced in ERM and MH eyes was used. In a comparison, the biomarker signature differed between the aqueous and vitreous from VR cases. Of note, a slight negative correlation was detected between the CP and FT levels in the aqueous (rho = −0.3419, *p* = 0.4528; Figure 5C), while no correlation was obtained for vitreous. Increasing trends were observed for IFN and tumour necrosis factor-α (TNF-α) (Th1) as well as for IL12p70 and IL10 (Th2) in aqueous with respect to vitreous humour from VR cases.

### 2.5. CP/FT Ratio and Drusen Association: Odds Ratio Analysis

Therefore, we aimed to examine these biomarkers’ ability and accuracy in identifying potential AD associations independently from clinical presentation. To better understand their function, we used MedCalc software for odds ratio (OR) calculation (OR values with upper and lower limits calculated with a 95% CI), starting with the study population, which was clustered depending on AD assessment and high CP and/or FT outcomes. The validity of the results obtained with this approach was tested by evaluating and comparing the obtained OR values. The above data display a specific CP and FT stratification depending on the disease severity. Using this approach, we observed high CP in a stage 2 case, which presented 75% of CP and FT (OR, 2.40; 0.30–19.40), in contrast to the stage 3 (OR, 1.50; 0.18–12.46) and stage 4 (OR, 0.86; 0.09–8.07) cases, which showed a percentage of variants of 8.3% and 16.6%, respectively. Since the stage 4 cases resulted mostly in OR > 1, we can conclude that, in our model, an increased occurrence of AD risk (risk response or positive association) was detected in stage 4 cases, although this requires further investigation.

### 2.6. CP/FT Validation in a Few AD-Selected Biological Samples

**Figure 6:**
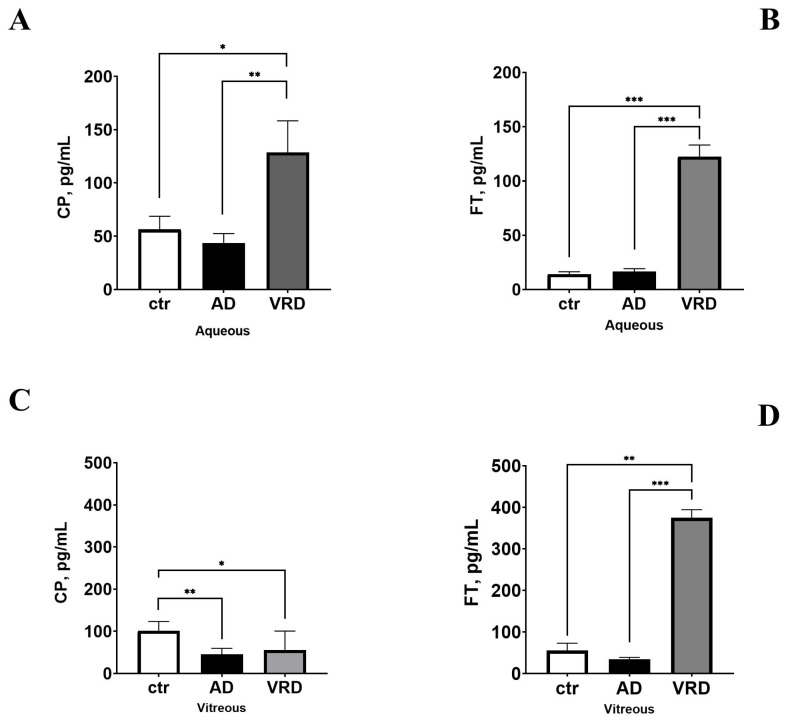
Ceruloplasmin (CP) and ferritin (FT) in ocular fluids from patients with AD-VRD. Histograms showing the different expressions of ceruloplasmin and ferritin, respectively, in aqueous (**A**,**B**) and vitreous (**C**,**D**) humours. Note the decreased levels of both proteins in aqueous and vitreous from patients with AD, compared to VRD groups. Legend: VRD, vitreoretinal diseases; AD, Alzheimer’s disease; ctr, normal control group. Data (mean ± SD) were from ELISA and compared with ANOVA Tukey-Kramer post hoc analysis. Specific *p* values are shown by asterisks (* *p* < 0.05; ** *p* < 0.01 or *** *p* < 0.005).

The presence of a few patients with AD who required therapeutic phaco-vitrectomy allowed us to verify the association between CP and FT in this population. In these subjects, the CP levels were significantly low in aqueous (12.73 ± 1.39 vs. 41.71 ± 14.43 pg/mL; AD vs. ctr; *p* < 0.05) and vitreous (12.27 ± 1.40 vs. 41.71 ± 14.43 pg/mL; AD vs. ctr; *p* < 0.05) fluid from patients with AD-VRD, as validated by ELISA (Figure 6A–C). As well, the FT levels were low in aqueous (12.73 ± 1.39 vs. 41.71 ± 14.43 pg/mL; AD vs. ctr; *p* > 0.05) and vitreous (41.71 ± 69.29 vs. 128.97 ± 4.35 ng/mg; AD vs. ctr; *p* < 0.05) fluid from AD-diagnosed patients with VDR, as validated by ELISA (Figure 6B–D).

**Figure 7:**
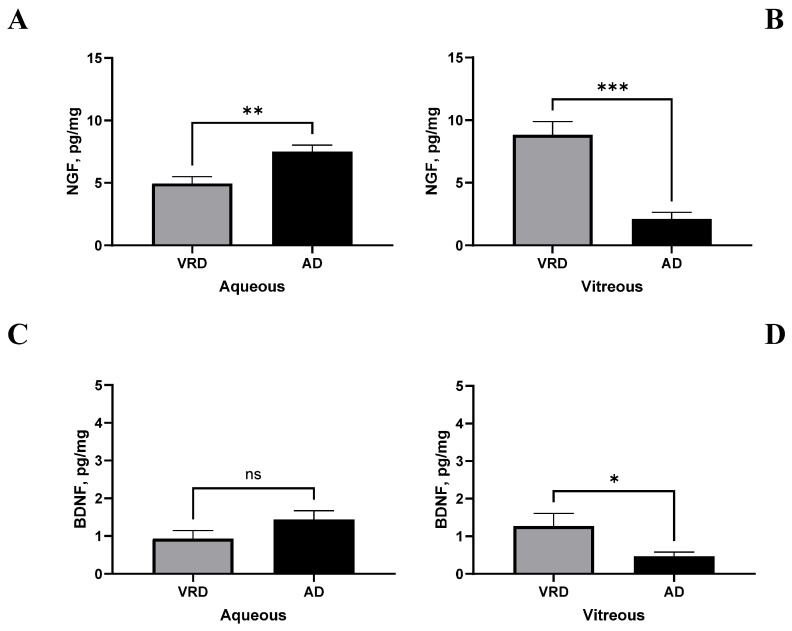
Neurotrophin content in ocular fluids from patients with AD-VRD patients. Histograms showing the different expressions of NGF and BDNF, respectively, in aqueous (**A**,**C**) and vitreous (**B**,**D**) humours. Note the increased levels of NGF in aqueous from patients with AD-VDR and the decreased levels of both NGF and BDNF in vitreous from patients with AD-VDR, compared to the lone VRD group. Legend: VRD, vitreoretinal diseases; AD, Alzheimer’s disease. Data (mean ± SD) were from ELISA and compared with ANOVA Tukey-Kramer post hoc analysis. Specific *p* values are shown by asterisks (ns not significant; * *p* < 0.05; ** *p* < 0.01 or *** *p* < 0.005).

According to the main role of neuroinflammatory mediators in neurodegeneration, we checked the relation between CP, FT, and a few neuromodulators. The two major neurotrophins, NGF and BDNF, recognized as well-known actors that take part in neurogenesis and neurodegenerative events, were investigated by microarray analysis and validated by ELISA. As shown in Figure 7, the NGF levels increased in the aqueous (2.128 ± 0.571 vs. 8.828 ± 1.138 pg/mg; AD vs. ctr; *p* < 0.01; Figure 7A) and significantly decreased in the VH (7.518 ± 0.729 vs. 4.967 ± 0.805 pg/mg; AD vs. ctr; *p* < 0.005; Figure 7B) of patients with AD-VRD with respect to controls. As well, the BDNF had a slight tendency to increase in the aqueous (1.441 ± 0.063 vs. 0.933 ± 0.381 pg/mg; AD vs. ctr; *p* > 0.05; Figure 7C) and to significantly decrease in the vitreous (0.471 ± 0.063 vs. 1.273 ± 0.381 pg/mg; *p* < 0.05; Figure 7D) of patients with AD-VRD with respect to controls. Both neurotrophins’ levels were assessed by conventional specific ELISA.

## 3. Discussion

The present study confirms the presence of CP and FT in ocular fluids under normal conditions and highlights their dysregulation in ERM and MH diseases. The alterations appear closely linked to disease severity and are associated with the expression of specific inflammatory (IFNγ, IL12p70, IL-6, IL4, and VEGF) and neurodegenerative (NGF, BDNF) mediators. The potential predictive value of CP and FT in AD-related macular conditions was further explored by evaluating the association between the CP/FT ratio and reported AD cases through odds ratio analysis. The pathological relevance of these findings is discussed below.

First, the CP and FT levels were measured in ocular fluids from normal (ctr) and pathological eyes (VRD). The baseline levels of CP and FT in normal ocular fluids were in line with previous studies carried out on control biological samples (aqueous and vitreous) [32]. Serum CP and FT are widely recognized as inflammatory biomarkers, and both proteins serve as indicators of cellular damage in conditions such as AD, dementia, and AMD [33]. Under physiological conditions, CP and FT are involved in the transport and homeostatic regulation of copper and iron between the bloodstream and peripheral tissues [32,33]. FT is only implicated in iron homeostasis [34]. An interesting functional aspect of these two proteins is related to cellular and tissue iron homeostasis [32]. The ability to protect the retina either in the case of oxidative stress or during trace metal accumulation has been described in experimental models [35]. The detection of CP and FT in ocular fluids described in this study provides an additional biological matrix for the quantification of these biomarkers, as also suggested by previous studies [35]. The ability to measure both proteins in samples obtained during phaco-vitreoretinal surgery opens promising perspectives for their application in predictive diagnostics and personalized therapeutic strategies.

Second, we tested whether CP and FT levels could be linked to VRD. No significantly increased levels of CP and unchanged levels of FT were observed in aqueous from patients with VRD with respect to control, while significantly decreased levels were observed for CP in the vitreous and a tendency to decrease was observed by an assay for FT in vitreal fluids. Their impaired expression in the vitreous would suggest: (i). a high permeability of the retinal vessels, (ii). a potential intracellular synthesis of both proteins, and/or (iii). the presence of concomitant inflammatory conditions [32,36,37]. The blood–aqueous barrier is a crucial component of the blood–ocular barrier (which also includes the blood–retinal barrier), since it regulates the movement of substances between the blood and the aqueous humour. Briefly, this low-viscosity fluid is synthesized by the cells of the ciliary processes in a three-step course (diffusion, ultrafiltration, and active secretion) and contains amino acids, electrolytes (sodium and potassium), oxygen, ascorbic acid, carbohydrates, glutathione, immunoglobulins, and organic and inorganic ions [38]. Physiologically, the aqueous turnover is an active process due to the dynamic secretion of fluid from the non-pigmented ciliary epithelial cells in the posterior chamber, movement to the anterior chamber through the pupil, and drainage out (90%) through the trabecular meshwork into the Schlemm’s canal [39]. Upon the activation of these well-organized tight junctions, small amounts of plasma-derived protein reach the aqueous by diffusion (from ciliary stroma to iris stroma) and are available at the anterior chamber, but the appropriate composition could be altered by disruption of the blood–ocular barriers (blood–aqueous barrier and the blood–retinal barrier; increased vascular permeability or microbial imbalance), leading to ocular inflammation, or, alternatively, by inflammation driving the disruption of blood–ocular barriers [40]. No less, the clear gel-like vitreous provides nutrients to the retina, the part of the eye that communicates with the brain [41]. Altered aqueous and vitreous compositions, as in VRD, as well as their impaired CP and FT content, as we observed, might impact the ability to link copper and iron, two metals involved in the functional activity of several enzymes that are necessary for matrix metabolism [35]. A reduced CP expression can lead to the accumulation of essential trace metals and inflammatory-driven tissue damage [42]. Since CP works as ferroxidase, CP primarily mediates the iron homeostasis (impaired metabolism) in the bloodstream and tissues, including the eye and retina [32]. Since FT is mainly involved in iron regulation, avoiding oxidative stress, and cell damage/death, the common CP and FT expression would suggest a shared contribution to the development of neurodegenerative disorders [43]. Due to their regulation of ECM homeostasis, these proteins have been suggested to be crucial for the retina, as previously observed in VRD and AMD, and, particularly, in the bloodstream of patients with AD and PD [44]. Regarding the cellular subsets involved in CP and FT production, we can hypothesize a crucial role for Müller cells, hyalocytes, astrocytes, retinal ganglion cells (RGCs), and some inflammatory cells, which populate the epiretinal membranes, the underneath stressed retina, and the reactive cells belonging to the inflamed vitreous [45]. Particularly, the dysregulation of CP expression by Müller cells has been reported for various retinal diseases, including AMD, diabetic retinopathy, and retinal detachment [46]. Finally, the expression of CP by Müller cells was associated with oxidative stress control, mostly due to the well-known antioxidant properties of CP (iron homeostasis) that protect Müller cells from oxidative stress and inflammation, two deleterious events that might occur in an autocrine fashion [45,47]. Lastly, aqueous and vitreous (salts, sugars, proteins, and collagen, as well as several resident and eventually inflammatory cells) fluid compete to guarantee the entire ocular homeostasis [48].

An interesting question underlies the CP and FT levels’ association with ERMs and MHs. Our findings on the altered expression of CP and FT in ERMs and MHs might be explained by a different grading of inflammation or disease severity. Recently, the two forms of CP were observed in the eyes of patients with high intraocular pressure and established glaucoma: the holo-CP (six copper atoms per molecule) and the apo-CP (the protein without the copper) [49]. CP is an acute-phase plasma protein with ferroxidase activities that is locally produced by activated monocytes/macrophages and retinal glia, and which is abundantly released in the ECM compartment upon response to inflammation, trauma, or infection [47]. Upon light damage, CP (retinal ferroxidase) is quickly upregulated in Müller cells, after which the protein amasses first locally and thereafter in the brain, while excessive CP protein accumulates in the vitreous [50]. The functional significance of this increase finds an explanation in the specific counteracting effects on oxidative stress, specifically the decrease in ferrous iron associated with ROS production [51]. These protective findings suggest that CP levels might influence the severity of retinal degeneration, especially at the initial stages of ERM [52]. On the contrary, CP might be the main factor responsible for the decreased amount of FT in aqueous and vitreous, contributing to the decrease in the protective effects of FT [52]. This aspect is supported by the observation of a relationship between local CP and FT, as detected by correlation analyses. The negative correlation would suggest a disequilibrium between these proteins and, likewise, the related trace metals. This raises the question of the major sources of aqueous and vitreous CP and FT upon inflammation. At the cellular level, ERMs and MHs are different: while ERMs involve membrane formation on the macula, MHs are holes in the macula [53]. Precisely, ERMs (macular pucker) refer to scar fibro-cellular tissue that origin on the macula under unknown stimuli (idiopathic ERM), physical or biological (injury, trauma, or pathological states such as uveitis or diabetes) insults, or simply ageing, with vitreous pull away from the retina causing tension and tears in the macula [54,55]. The pathological changes that occur in the vitreous will be reflected in the morphology and function of the underlying retina, and these processes might originate from sources outside the vitreous [27]. Senescence, ageing, chronic inflammation, and mitochondrial damage can sustain long-lasting oxidative stress, which in turn can activate nitric oxide synthase and the formation/accumulation of reactive oxygen species/reactive nitrogen species (ROS/RNS) [56]. The impairment of metal tracers and their specific carriers can provide support to drusen deposits and retinal impairments in both ERMs and MHs [57]. Our finding prompts an association of CP and FT with retinal drusen build-up, but the knowledge about the cellular mechanisms behind the initiation and deposition of drusen inside the retina is still very limited [58].

An interesting point lies in the possibility of understanding if CP and FT levels are linked to systemic inflammatory, metabolic, or neurodegenerative targets. An important aspect of this and previous studies is the definition of an ocular fluid protein signature that makes it possible to monitor, for instance, treatments in ERM and MH [59]. Several growth factors—TGFβ, VEGF, angiotensin 1 and 2 (Ang-1/Ang-2), neurotrophins (NGF and BDNF), neuropeptides (NPY and VIP), and the overstudied inflammatory molecules (Th1/Th2 path)—are released upon any kind of retinal insult and drive the migration of retinal cells like from retinal pigmeted epithelium (RPE) to the vitreous to proliferate and induce retinal membrane formation and drive contraction/retraction at the vitreoretinal interface [60]. First, we observed that that association between CP and FT occurred with a few selected inflammatory (IFNγ, IL12p70, IL6, IL4, and VEGF) and neurodegenerative (NGF, BDNF) mediators. While VEGF represents the major angiogenic mediators, the others, IFNγ, IL6, and IL12p40, might represent accessory mediators [61]. The expression profiles of VEGF and TGFβ did not show a specific relationship with CP and/or FT. Another aspect is related to CP and IL6, well-known multifunctional cytokines with proinflammatory and angiogenic functions that mainly occur through the induction of VEGF inside the retina [62]. Of interest, the association between CP and IL4 might open to additional hypotheses to support the involvement of CP at the vitreal compartment [52]. The persistence of vitreomacular traction in ERMs and, later, the formation of MHs might be supported by the CP and FT vitreal signature and its association with pro-fibrogenic and pro-angiogenic mediators [32].

An interesting point is related to the possible use of the CP/FT ratio and drusen imaging association for an early diagnosis of neurodegenerative states. The odds ratio analysis helped us to associate the CP and FT levels with diagnosed AD in patients with VRD. Despite the limitation of its size, our study population displayed the presence of drusen formations in most VRD cases, and these were mainly localized in the choroidal-macular area. Drusen formations are common to ERMs and MHs, as well as other neurodegenerative ocular diseases, but they do not point to the existence of a neurological assessment [8]. CP and FT changes in association with retinal drusen build-up, taped by OCT, might represent an additional link with the early diagnosis of neurodegenerative disorders, depending on the size of the deposits [63]. This CP and FT impairment, as well as the association of CP and FT with drusen formations, could be explained by their tight relation with the choroid structure and increased vessel formation [64]. Indeed, the abnormal expression of these two metal carriers in association with a diagnosis of AD would reinforce the potential predictive ability of these markers in ocular fluids in the case of differential diagnosis (odds ratio). It is noteworthy to discuss that the accumulation of Aβ protein in AD brains and eyes has been suggested to induce neurodegeneration, especially in the hippocampus, leading to the progressive loss of cognitive function [12]. Postmortem human retinal tissues showed Aβ deposits above the RPE and located in the inner retina, which is associated with neuronal loss, and were especially localized within the ganglion cell layer (GCL), inner nuclear layer (INL), and inner plexiform layer (IPL) districts [12]. Although not investigated herein, a small amount of evidence supports the observation that heavy metals might be involved in the pathogenesis of AMD [65]. Beyond the deposition of copper, iron, and zinc deposits in AMD, recent studies have shown the accumulation of heavy intoxicant metals in the eye tissues and bloodstream of patients with AMD [66]. The CP/FT validation in a few AD samples might represent a starting point in prospecting an association between VRD and neurodegenerative AD events. Posterior vitreous detachment is part of the normal process of ageing and involves specific changes to the cellular and soluble components of ocular fluids [27].

Several limitations of this study should be acknowledged. First, the pilot nature of this study and the relatively small sample size—predominantly composed of patients in advanced stages of disease—may introduce selection bias. Additionally, the cohort included only patients with a confirmed diagnosis of AD, while those with mild cognitive impairment (MCI) or lacking a comprehensive neuropsychological assessment, as well as positron emission tomography (PET) and magnetic resonance imaging (MRI) imaging data, were not included. Despite these limitations, the analysis of ocular fluids remains a valuable approach for exploring disease pathophysiology and assessing treatment responses in a variety of ocular and potentially neurodegenerative conditions [67]. However, the aqueous consists of very small and limiting volumes, meaning that its translation into routine diagnostics presents difficulties in compared to analysis with traditional ELISA techniques, while the vitreous has the right volume but is accessible only in the case of vitreoretinal surgery, which limits the possibility of using it as a diagnostic rule [68]. Finally, increased levels of CP and FT in ocular fluids would imply the presence of a dysregulation of the free tracers available in the tissues. In a previous study, it was theorized that the low serum levels of CP coupled with high iron levels led to retinal degeneration, as CP might help to prevent the oxidative damage caused by ferrous iron by oxidizing iron from its ferrous to ferric form [52]. Copper or iron measurement could offer valuable insights into metal-related pathogenic mechanisms and further clarify the relationship between trace metal homeostasis and neurodegenerative or retinal processes.

Taken together, our finding extends previous studies on the topic and implement our knowledge on the usefulness of ocular fluids as a biological matrix for the diagnosis of neurodegenerative diseases. As is known, the clinical spectrum of VR disorders ranges from pure to neurodegenerative-associated forms of ERM and MH, which includes AMD and CNV, which range dynamically from asymptomatic and MCI to mild, moderate, or severe states of activation [69]. The primary clinical measures for assessing AD progression are still neuropsychological tests, neuroimaging biomarkers, and a few selected plasma biomarkers with high diagnostic performance [70,71]. In this study, we explored and tested the hypothesis that ocular fluids represent an alternative, accessible biological matrix for biomarker detection. Our patient cohort, composed of individuals with ERMs and MHs who were undergoing vitreoretinal surgery, exhibited varying degrees of drusen formation (as assessed by SD-OCT) and distinct CP and FT expression profiles. These patterns suggest a potential association between ocular CP/FT levels and AD-related processes. Although the observed differences did not reach statistical significance, the findings support the rationale for further investigation in larger, well-characterized populations.

## 4. Materials and Methods

### 4.1. Study Population and Ethical Considerations

This is a single-centre and prospective study which studied a population including 26 patients (18F/8M; 71.45 ± 7.36 years old) with vitreoretinal interface diseases that were consecutively selected for vitrectomy coupled to cataract removal. This study was approved by the intramural ethical committee (IFO-Bietti, Rome, Italy) and carried out in accordance with the ethical standards stated in the Declaration of Helsinki. Patients approved the experimental protocol and signed the informed consent for ocular examination and the collection, handling, analysis, and storage of residues of specimen.

As inclusion criteria we considered the diagnosis of epiretinal membranes and macular holes and the necessity of therapeutic cataract. The presence of any potential factor that indicated increased baseline inflammation or eye anatomy that could influence the surgery was a criterion for exclusion (diabetic retinopathy, retinal vein occlusion, ocular inflammatory disease, trauma, intraocular surgery, intraocular tumours, and retinal tear or detachment). A full ophthalmic examination was carried out before surgery, including anamnesis, funduscopic evaluation, and spectral domain-optical coherence tomography (Spectralis SD-OCT ver. 1.5.12.0; Heidelberg Engineering, Heidelberg, Germany). At the recruitment ocular examination, patients were interviewed for regress and presentation of ocular and systemic diseases. During the visit, some subjects referred to an association with cardiological and neurological (Alzheimer diseases, Parkinson’s diseases, and senile dementia) diseases, and some patients also referred to hypercholesterolemia (Table 1). The accurate ophthalmological examination was implemented with a revision of treatment history and systemic diseases including Alzheimer, multiple sclerosis, and dementia. Exclusion criteria included proliferative diabetic retinopathy, age-related macular degeneration, retinal vascular occlusion, aphakia, high myopia (more than 28 diopters), or uncontrolled glaucoma. Additional exclusion criteria were prior vitrectomy, intravitreal injections, retinal laser photocoagulation, or opacity of optical media that would have disturbed fundoscopic evaluation or SD-OCT imaging.

### 4.2. Sampling and Preanalytical Procedure

At the time of surgery, vitreous and aqueous were sampled. Vitreous was collected at the beginning of pars plana vitrectomy, just before opening the infusion port, while ERM was next peeled off. The procedure of sampling included the vitreous collection at the beginning of standard 25 G pars-plana vitrectomy, just before opening the infusion port. Vitreous (250–500 μL) was quickly centrifuged (2000 rpm for 7 min; Sigma 1–14 microfuge, Merck SA, Consociata di Merck KGaA, Darmstadt, Germany) to separate floating cells from clarified supernatant that was quickly stabilized with protease inhibitors (1 µL/sample; Pierce, Thermo Fisher Scientific, Waltham, MA, USA), sonicated (VibraCell; Sonics, Newtown, CT, USA), and further centrifuged to remove debris (13,000 rpm/7 min). A spectrophotometer analysis (3 μL) was carried out (N1000, Nanodrop; Celbio, Euroclone S.p.A, Milano, Italy) before aliquots devoted to biochemical analysis were produced (Protein Chip Array and ELISA). ERMs were fixed in Thinprep (Hologic, Inc., Marlborough, MA, USA) and thereafter lysed for protein or RNA extraction.

### 4.3. ELISA

Ceruloplasmin, ferritin, NGF, and BDNF were measured by conventional ELISA (ceruloplasmin: code EIACPLC; Thermo Fisher Sci.; ferritin: code ab108698; Abcam, Cambridge, UK; code DY256-05 NGF and code DY248 BDNF: duo-set kits from R&D System, Minneapolis, MN, USA). Vitreous and aqueous were prediluted (1:2) in lysis buffer supplemented with protease inhibitors (PMSF; Merck SA, Consociata di Merck KGaA, Darmstadt, Germany) and loaded on 96-well precoated plates. Standard curves and steps in the procedure were carried out according to the manufacturers with minor modifications. Standard curve ranges and detection limits were set according to the manufacturers. Specific OD values were recorded after reading the plates at λ490 nm corrected to λ560 nm in a 96-well plate reader (Sunrise; Tecan, Männedorf, Switzerland), and the single data were obtained by using the polynomial third-order function (r = 0.999) generated and used in graph pad.

### 4.4. Protein Array

The concentration of Cytokines was measured by means of a customized protein array on glass-chips from RayBiotech™ technology, according to the manufacturer’s instructions (Norcross, GA, USA). Vitreous and aqueous were prediluted (1:2) in lysis buffer supplemented with protease inhibitors (PMSF; Merck SA) and loaded on chip array precoated plates. Glass slides were processed by GenePix 4400 Microarray scanner (Molecular Devices LLC, Sunnyvale, Silicon Valley, CA, USA). Fluorescence signals were acquired with a GenePix 4100 microarray scanner (Molecular Devices LLC, Sunnyvale, CA, USA) equipped with the GenePix pro 3.0 software (Axon Instruments, Foster City, CA, USA). An inter- and intra-assay coefficient of variability limit of ≤10% was set, and a 1.5-fold increase or ≤0.65-fold decrease in signal intensity was considered to guarantee specific signals above background. Fluorescent signals were analysed and fold changes were generated (pathological/control ratio). In order to minimize intra- and inter-assay variability, a single tester handled all the material and followed all experimental phases.

### 4.5. Statistics

Subjects’ data were reported in Excel files and then transferred to the Prism10 platform (GraphPad Prism v.10.4.2 software Inc., La Jolla, CA, USA). The Kolmogorov–Smirnov Test was preliminarily run to assess the normality of the sample distribution (descriptive analysis) and the subsequent choice of the appropriate statistical test. For clinical comparative analysis, 2 groups were produced (control and case), whose clinical data were analysed by linear regression and *t*-test. Biomolecular data were analysed by linear regression and variance analysis (ANOVA). The significance limit was set at *p* < 0.05 (95% confidence value). The MedCalc ver. 22.021 for Windows 11 64-bit (MedCalcSoftwareLtd© 2024, Ostend, Belgium) was used for odds ratios (ORs) and Chi square calculations. Results were indicated in the text as OR values with low and high limits according to the 95% confidence intervals (CIs). As a rule, the ORs less than 1 favoured this cluster model. Panels in Figure 5 were created using the GraphPad Prism 9.4 software (GraphPad Software; San Diego, CA, USA).

## 5. Conclusions

Proteomics-based research is now uncovering dynamic brain changes that occur during Alzheimer’s progression, offering up potential therapeutic targets. AD dementia refers to a particular onset and course of cognitive and functional decline that is associated with age, together with a particular neuropathology. AD is now the most common form of neurodegenerative dementia worldwide [72]. Recently, CP (copper storage) and FT (iron storage) have been associated with AD, although this remains debated in preclinical AD, before the onset of cognitive impairment not related to dementia [73]. CP is one of the two extracellular ferroxidases that convert ferrous iron into ferric iron, providing tissue protection from iron toxicity, as in the case of excessive copper and iron inside the brain and retina [52]. VRD forms are conditions that affect the posterior chamber, causing the progressive loss of central vision with the dysregulation of para-inflammation, the innate response (complement), and inflammatory, angiogenic, and ECM clusters [74]. By 2020, the VRD population is expected to increase vertiginously, and this disease represents one of the major public health problems, having substantial socioeconomic implications [74]. New predictive biomarkers of AD and dementia, as well as VRD, might help in achieving the early predictive and accurate diagnosis of the disease. The use of the copper/ceruloplasmin ratio (Cu:Cp) has been proposed to overcome such problems [72,74]. Herein, we demonstrated that the quantification of CP and FT coupled to specific expressions of some inflammatory and neurotrophic mediators can be possible in ocular fluids and sediments in a non-invasive manner, and can be used in association with OCT imaging and coupled to routine tests for AD diagnosis. Moreover, we prospect the potential use of CP and FT detection in the ocular fluids of patients with VRD as a non-invasive method of screening in patients with familial linkage.

## Figures and Tables

**Table 1:** Table summarizing demographic characteristics (A) of the study population and the stratified comorbidities (B) as declared by patients at the time of complete ocular visit for enrolment (past and present anamnesis). Vitreoretinal samples collected at the time of surgery are in brackets. The optical coherence tomography (OCT)-based classification [25] and the OCT-based classification of the International Vitreomacular Traction Study Group for the classification of full-thickness macular holes (MHs) were used for the classification of epiretinal membranes (ERMs) and macular holes (MHs), respectively. Legend: ERMs, epiretinal membranes; MHs, macular holes; AD, Alzheimer disease; M/F, male/female; data (average age) are expressed as mean ± standard deviation (SD). *p* values were according to the *t*-test analysis.

Study Population	Cases	ERMs	MHs	AD	*p* Value
A. demographic characteristics					
Participants/[biosamples]	26/[26]	14/[14]	6/[6]	6/[6]	
mean age	69.60 ± 6.71	70.00 ± 6.60	68.70 ± 7.00	70.00 ± 6.60	*p* = 0.385
sex	8M/18F	3M/11F	2M/4F	3M/3F	*p* > 0.05
ERM stage 2		1M/5F			
ERM stage 3		1M/4F			
ERM stage 4		2M/1F			
B. comorbidities					
Hypertension	19/26	10/14	6/6	5/6	
Hypercholesterolemia	11/26	7/14	2/6	2/6	
Cardiopathy	5/26	3/14	2/6	2/6	

## Data Availability

All data are included in the manuscript.
